# What Moves Men to Change? A Mixed‐Methods Study on Facilitators and Barriers of Lifestyle Changes in Men Seeking Fertility Care

**DOI:** 10.1111/andr.70133

**Published:** 2025-10-26

**Authors:** Wiep R. de Ligny, Kathrin Fleischer, Hilde Grens, Didi D. M. Braat, Inger L. Abma, Jan Peter de Bruin

**Affiliations:** ^1^ Department of Obstetrics and Gynaecology Radboud University Medical Center Nijmegen the Netherlands; ^2^ Nij Geertgen Center for Reproductive Medicine Elsendorp the Netherlands; ^3^ Center for Reproductive Medicine, Jeroen Bosch Hospital ‘s‐Hertogenbosch the Netherlands; ^4^ IQ Health Science Department Radboud University Medical Center Nijmegen the Netherlands

**Keywords:** lifestyle, male fertility, obesity, patient‐centeredness, smoking

## Abstract

**Background:**

An unhealthy lifestyle negatively affects male fertility. Despite this, men that are part of an infertile couple often fail to improve their lifestyle and evidence on influencing factors is limited.

**Objectives:**

To identify facilitators and barriers involved in lifestyle changes of men seeking fertility care and in lifestyle counseling by fertility health care providers (HCPs).

**Materials and Methods:**

A mixed‐methods study was performed including semi‐structured interviews with 14 men seeking fertility care and seven fertility HCPs. Fifty other men completed a questionnaire evaluating various aspects of lifestyle change. Eligible participants were men part of an infertile couple and met at least one lifestyle criterion: BMI ≥ 25 kg/m^2^, smoker, alcohol use of ≥ 7 units/week, and recreational drug use. Included HCPs provided lifestyle counseling to infertile couples.

**Results:**

The most important facilitators for lifestyle changes in men seeking fertility care are the wish to improve their chances to father a child and their partner's support. The most important barriers are stress, a busy life, an unhealthy lifestyle being part of social activities, and normal semen quality. HCPs experienced limited time, unclear and insufficient scientific evidence on lifestyle and male infertility, and lack of uniform care as barriers. Professional responsibility and societal factors were facilitators.

**Discussion:**

HCPs could use these results to improve and personalize lifestyle counseling of men seeking fertility care. For example, by emphasizing the impact of lifestyle on pregnancy loss and offspring in men with normal semen quality. This study is limited by its small sample size and its confinement to a Dutch context.

**Conclusion:**

This study identifies previously unknown facilitators and barriers for lifestyle changes in men seeking fertility care. It also reveals barriers experienced by HCPs when counseling male patients about lifestyle. These results should inspire fertility departments to (re)design lifestyle interventions and policies for men seeking fertility care.

## 1 Introduction

Infertility is defined as the inability to conceive after 1 year of regular and unprotected intercourse. Globally, approximately one in every seven couples faces infertility, and in about half of them a male factor is involved [1, 2, 3]. Treatments to improve male fertility are limited, frequently leading health care providers (HCPs) to resort to assisted reproductive technology (ART). Infertility and ART treatment are associated with significant psychological stress and incur substantial health care costs [4, 5].

Given these challenges, it is crucial to explore alternative methods to improve male fertility. Research consistently indicates that an unhealthy lifestyle adversely affects male fertility [6]. Even more concerning is its potential impact on future offspring, including mental and behavioral problems [6, 7, 8].

Paternal obesity has been linked to low sperm quality, poor ART outcomes, and an increased risk of pregnancy loss [9, 10, 11]. Smoking is known to impair sperm quality and is associated with a higher risk of pregnancy loss and birth defects [12, 13, 14, 15, 16, 17, 18, 19]. Although alcohol consumption has been less studied in men seeking fertility care, a decrease in live births following IVF has been observed when both partners consume alcohol [20]. Chronic alcohol consumption causes a different hormonal profile and lower semen parameters [21].

Cannabis use is associated with impaired semen quality, erectile dysfunction, and testicular atrophy, but evidence is inconsistent [22, 23]. Paternal use of opioids may influence offspring behavior and neurodevelopment [24]. Despite increasing use, research on the influence of cocaine and methamphetamines on male fertility is limited. Animal studies indicate that parental exposure to methamphetamines can cause epigenetic changes in offspring, and that paternal cocaine exposure alters offspring behavior [25, 26].

Studies show that these adverse effects on male fertility can be reversible. Loss of abdominal fat resulted in less sperm DNA damage and an improvement in hormonal profiles [27]. Diet‐induced weight loss led to improved semen parameters in men with obesity [28, 29]. There are no studies on the reversibility of the effect of tobacco, alcohol, and recreational drug use in men.

Considering the available evidence, successful and durable lifestyle changes in male fertility patients could improve semen quality and pregnancy rates. This could lower the need for ART, thereby decreasing health care costs. Other positive effects on men, such as improved mental health and lower risk of cardiovascular disease, also make this an attractive treatment option.

As is widely recognized in clinical practice, men seeking fertility care often fail to improve their lifestyle. Clearly, this is important for men with impaired semen quality, as lifestyle improvement could improve semen quality and their chance to father a child. But it is also relevant for men with normal semen quality, as it can lower the risk of pregnancy loss and the effect on future offspring [12, 13, 14, 15, 16, 17, 18, 19].

The aim of this study was to identify facilitators and barriers for lifestyle change in men seeking fertility care. We also assessed factors involved in lifestyle counseling of men by fertility HCPs. The findings of this study will provide new insights into improving lifestyle counseling and support for men seeking fertility care.

## 2 Methods

### 2.1 Study Design

This mixed‐methods study includes two parts: a qualitative part consisting of semi‐structured interviews with patients and HCPs, and a quantitative part consisting of a patient questionnaire.

### 2.2 Study Setting

This study was conducted at the department of reproductive medicine of a non‐academic teaching hospital (Jeroen Bosch Hospital), an academic center (Radboudumc), and a private fertility clinic (Nij Geertgen Fertility Clinic), all located in the Netherlands. Interviews were conducted between November 2022 and February 2023 and questionnaires were obtained between January and September 2024.

This study was not subject to the Dutch Medical Research Involving Human Subjects Act (WMO). A written confirmation that the WMO does not apply to this study was issued by the local Medical Ethics Committee Brabant.

### 2.3 Eligibility Criteria and Sampling of Patients

Eligible men were cisgender and in a heterosexual relationship, aged between 18 and 50 years, and referred to or under treatment at the department of reproductive medicine of one of the three participating centers. Considering the risks of an unhealthy lifestyle for all men seeking fertility care, the study population was not restricted to men with male factor infertility. Patients should meet at least one of the following lifestyle criteria: BMI ≥ 25 kg/m^2^, current smoker, current alcohol use of ≥ 7 units/week [20], current recreational drug use. Exclusion criteria were: treatment with cryopreserved or donor semen, and a known genetic or hormonal cause of infertility.

For the interviews, participants were included until data saturation was reached. Purposive sampling was applied, aiming for variation in lifestyle factors, age, and fertility diagnosis.

For the questionnaire, we aimed to include a separate group of 50 men. This sample size was estimated to be able to establish a hierarchy among the different facilitators and barriers.

### 2.4 Eligibility Criteria and Sampling of Health Care Providers

HCPs were required to work in fertility medicine and have regular interaction with men who are part of an infertile couple. Purposive sampling for HCPs was focused on age, sex, years of experience in fertility care, place of work, and profession. As urologists do not provide standard fertility care in the Netherlands, they were not included in the interviews.

### 2.5 The Interviews

#### 2.5.1 Data Collection

Patients were recruited by their fertility HCP after assessing eligibility. All interviews were conducted by the coordinating researcher, a female PhD candidate, MD (Wiep R. de Ligny) and were audio recorded. Patients were interviewed face‐to‐face at home or at the clinic, or via a secure video connection. All interviews were semi‐structured with use of a pre‐defined interview guide and lasted between 30 and 45 min. We anticipated that some of the interviewees had already initiated lifestyle changes, which then provided the interview context and guided questions such as: “What motivated you to change your lifestyle?” and “How did you maintain this change?”

HCPs were recruited by the coordinating researcher of the study and were all conducted face‐to‐face at the clinic. All participants signed written informed consent before the interview was conducted. Interview guides were created based on expert input from the study team.

#### 2.5.2 Data Analysis

The interviews were transcribed verbatim and analyzed by inductive content analysis. This means that no pre‐defined codebook was used, but codes and categories were constructed from the data through an iterative process. Relevant passages from the interviews were selected and coded with terms as close to the participant's words as possible. Coding and analysis of the first four patient‐ and HCP interviews were done by two researchers (Wiep R. de Ligny and H. Grens) independently and disagreements were discussed until consensus was reached. Factors were identified, grouped into categories, and discussed within the study team for further refinement. Next, all interviews were searched for these newly defined factors and categories. Because the patient interviews and the HCP interviews had a different research question, two distinct codebooks were defined and applied. The COREQ guideline was used to report the qualitative research [30].

### 2.6 The Questionnaire

#### 2.6.1 Content of the Questionnaire

The questionnaire included three sections. In the first section, patients were instructed to rank the facilitators and barriers identified in the interviews according to personal importance. Patients were requested to select between one and five facilitators and barriers.

The second section consisted of a selection of relevant questions from the Patient Centeredness Questionnaire Infertility (PCQ‐I) [31]. The selected and adjusted questions are presented in the Results section and were used to assess the role of the HCPs in participants’ lifestyle changes.

In the third section, intention to change lifestyle was assessed on a 5‐point Likert scale (ranging from very unlikely to very likely). Patients were asked the following question: “Are you planning to [change your lifestyle in a specific way] within the next 6 months?” This tool was previously validated for smoking cessation [32].

Before dissemination, the questionnaire underwent one round of cognitive validation by discussing the questionnaire with three participants who had taken part in the interviews. Based on the patients’ feedback, minor changes were made to the lay‐out of the questionnaire, and to the order and wording of questions.

#### 2.6.2 Data Analysis

For the rating of the facilitators and barriers, we defined which factors were most frequently chosen as most important (number one) and reported those with a minimal frequency of 10%. Additionally, points were attributed to the most frequently chosen facilitators and barriers: five points if rated first, four points if rated second, etcetera. If a facilitator or barrier was not endorsed by a participant, it was attributed zero points. The mean rating per factor was calculated as the sum of the points, divided by 50 (participants). This method was considered appropriate to provide a descriptive overview of the different factors and their relative importance, but was not suitable to make statistical comparisons.

To test for differences in intention to change between different fertility diagnoses and lifestyle factors, we conducted an unpaired *t*‐test or a one‐way anova depending on the number of categories, using an alpha of 0.05 [33]. Pearson's *r* was calculated to evaluate to what extent male age and the duration of infertility correlated with the intention to change.

## 3 Results

### 3.1 Patient Interviews

#### 3.1.1 Patient Characteristics

Fourteen men were interviewed, with a median age of 37.5 years (Table 1). Most couples were diagnosed with unexplained infertility (42.9%), followed by male factor infertility (35.7%) and female factor infertility (21.4%). None of the couples had combined factor infertility. Median duration of infertility was 23 months. In the Netherlands, couples can be referred to a fertility clinic when they have failed to conceive within 12 months. Depending on the diagnosis, they are then assigned either expectant management for 6–12 months, or treatment with ovulation induction, intra‐uterine insemination, IVF, or ICSI. Insurance fully covers the first three IVF/ICSI cycles and all other fertility treatments.

**TABLE 1:** Characteristics of included patients and fertility health care providers.

Characteristics of interviewed patients (*n* = 14)	
Age, years, median (IQR)	37.5 (35.3–40)
Fertility treatment	
Expectant management/no treatment, *n* (%)	1 (7.1)
Intra‐uterine insemination (with or without hormones), *n* (%)	4 (28.6)
IVF, *n* (%)	4 (28.6)
ICSI, *n* (%)	5 (35.7)
Duration of infertility, months, median (IQR)	23 (18–28)
Fertility diagnosis	
Unexplained infertility, *n* (%)	6 (42.9)
Female factor infertility, *n* (%)	3 (21.4)
Male factor infertility, *n* (%)	5 (35.7)
BMI, kg/m^2^, median (IQR)	27.5 (25–31.3)
≥ 25, *n* (%)	9 (69.2)
Smoker, yes, *n* (%)	5 (35.7)
Alcohol consumption ≥ 7 units/week, yes, *n* (%)	8 (57.1)
Recreational drug use, yes, *n* (%)	2 (14.3)

Characteristics of interviewed fertility health care providers (*n* = 7)	
Age, years, median (IQR)	45 (40–49)
Experience in fertility care, years, median (IQR)	16 (8–20)
Sex	
Female, *n* (%)	5 (71.4)
Male, *n* (%)	2 (28.6)
Profession	
Fertility nurse, *n* (%)	2 (28.6)
Fertility clinician, *n* (%)	2 (28.6)
Gynecologist, *n* (%)	3 (42.9)
Place of work	
Academic hospital, *n* (%)	1 (14.3)
Non‐academic teaching hospital, *n* (%)	3 (42.9)
Private fertility clinic, *n* (%)	3 (42.9)

Characteristics of patients who completed the questionnaire (*n* = 50)	
Age, years, median (IQR)	32.5 (29–37)
Level of education^a^	
Low	5 (10)
Middle	22 (44)
High	23 (46)
Fertility treatment	
Expectant management/no treatment/do not know, *n* %	23 (46)
Intra‐uterine insemination (with or without hormones), *n* (%)	9 (18)
Ovulation induction, *n* (%)	7 (14)
IVF, *n* (%)	3 (6)
ICSI, *n* (%)	8 (16)
Duration of infertility, months, median (IQR)	24 (18–45.75)
Fertility diagnosis	
Unexplained infertility, *n* (%)	18 (36)
Female factor infertility, *n* (%)	13 (26)
Male factor infertility, *n* (%)	10 (20)
Combined factor infertility, *n* (%)	5 (10)
Unknown, *n* (%)	4 (8)
BMI in kg/m^2^, median (IQR)	28.1 (24.3–33.4)
≥ 25, *n* (%)	29 (58)
Smoker, yes, *n* (%)	18 (36)
Alcohol consumption ≥ 7 units/week, yes, *n* (%)	29 (58)
Recreational drug use, yes, *n* (%)	7 (14)

^a^ High, higher professional education or university; Low, primary or lower vocational education; Middle, secondary or intermediate vocational education.

#### 3.1.2 Facilitators and Barriers

**FIGURE 1:**
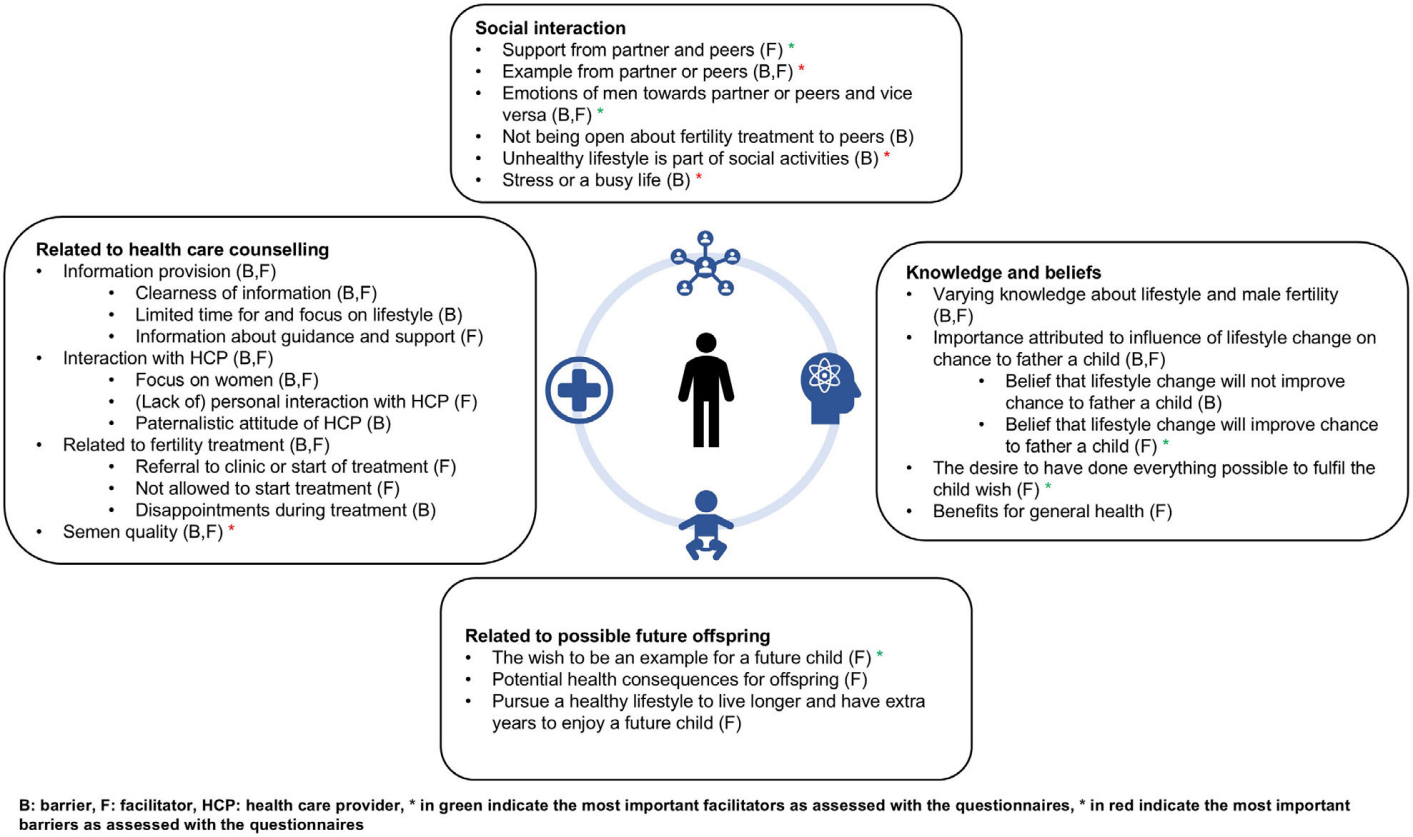
Facilitators and barriers of lifestyle changes in men seeking fertility care.

Three main categories of factors were identified for both the facilitators and barriers: (1) social interaction, (2) health care counseling, and (3) knowledge and beliefs about lifestyle and male fertility. For the facilitators a fourth main category was found: factors related to possible future offspring (Figure 1).

##### 3.1.2.1 Facilitators and barriers related to social interaction

Common facilitators were the support from partner and peers, and their feeling of disappointment, “[My friends] repeatedly told me, if you don't manage [to stop using drugs] now, we will call [professional help].” (L01), “When [my family and partner] are disappointed, it moves me. That has motivated me as well.” (L04). Some were motivated by the healthy lifestyle of their loved ones.

One of the barriers was not willing to be open about fertility treatment to peers. Men also frequently mentioned unhealthy habits being part of social events as a barrier, “everywhere we come, people are drinking alcohol.” (L07). Some reported fear of their peers’ reaction if they would decline smoking or consuming alcohol. Many interviewees experienced stress and lack of time as a barrier, although this was generally unrelated to the fertility problem.

##### 3.1.2.2 Facilitators and barriers related to health care counseling

Information provision can be a facilitator and barrier depending on the content. Although all participants had been informed about the effects of lifestyle on fertility by their fertility HCP, some regarded this information as too vague and not tailored to their personal situation, “HCPs tend to tell their patients ‘if you do this and that, everything will be fine’, but I think people feel more motivated if […] you address their problem more personally.” (L06). In line with this, mentioning specific numbers and percentages, and discussing options for guidance and support, were reported as facilitators.

The focus on women in fertility care was a facilitator for some men, “For me it is a small step compared to what my girlfriend has to experience, so I actually do not mind [to change my lifestyle].” (L08). However, it could also make men feel as if they are of less importance, which was a barrier to change. Other barriers were a paternalistic or coercive attitude and a lack of a personal approach by their HCP, “Like the doctor is telling the same old song.” (L06).

Referral to the fertility clinic, the start of fertility treatment, and if patients were not allowed to start treatment before lifestyle improvement, were reported facilitators. The disappointments related to fertility treatment were experienced as a barrier.

Impaired semen quality was often a facilitator, “It is pretty scary if the first test shows that your semen quality is not okay.” (L07). Yet some of these men felt no motivation to change if their semen quality was sufficient for fertility treatment. Normal semen quality was a barrier, because these men perceived that improving their lifestyle would not benefit their chance to father a child.

##### 3.1.2.3 Facilitators and barriers related to knowledge and beliefs

Knowledge and beliefs about lifestyle and male fertility often posed a barrier to lifestyle change. To about one‐third of the patients, the relationship between lifestyle and male fertility was not clear.

Another third indicated that they did not believe changing their lifestyle would improve their chance to father a child, “[My HCPs] are not going to guarantee that if I quit [cigarettes] or eat differently, that everything will be OK.” (L01). The most common argument for this belief was that people with an unhealthy lifestyle also have children, “When I look at people that had many children while they smoke so much, they also had children in the end.” (L05).

Many interviewees, especially smokers, were motivated to improve their lifestyle because of the benefits for their general health.

##### 3.1.2.4 Facilitators related to possible future offspring

Factors related to possible future offspring were all facilitators for change. Men wanted to be a good example for their future child, “I want to be a nice and cool father and not a lazy father that lies on the couch with a bag of crisps.” (L04); and were aware of the health risks for offspring, “Think about the health of a future child […]. Smokers get smaller children.” (L09). One patient mentioned the wish to live longer to spend more years enjoying the time with his child.

### 3.2 HCP Interviews

#### 3.2.1 Characteristics of HCPs

Seven fertility HCPs were interviewed about their experienced barriers and facilitators in lifestyle counseling of male patients (Table 1). The median years of experience in fertility care was 16 (interquartile range 8–20).

#### 3.2.2 Facilitators and Barriers

**FIGURE 2:**
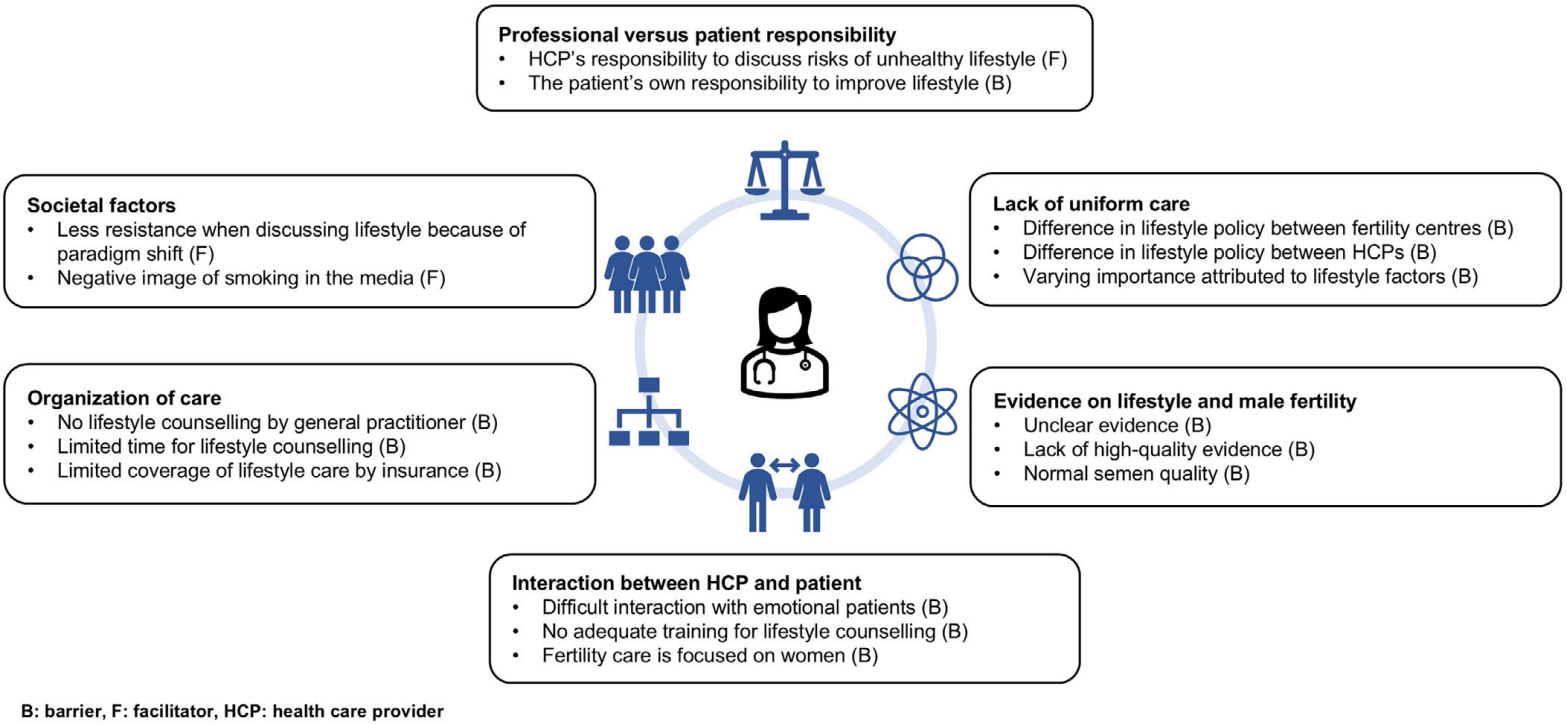
Health care provider‐reported facilitators and barriers for lifestyle counseling of their male patients.

Six categories were identified in the HCP interviews: (1) professional versus patient responsibility, (2) lack of uniform care, (3) limited and unclear evidence on lifestyle and male fertility, (4) interaction between HCP and patient, (5) organization of care, and (6) societal factors (Figure 2).

##### 3.2.2.1 Professional versus patient responsibility

All interviewees debated the balance between the responsibility of the HCP on one hand, and the responsibility of the patient (couple) on the other hand. The HCP's responsibility to inform male patients about the effects of an unhealthy lifestyle was experienced as a facilitator. Whereas the patient's own responsibility to improve their lifestyle proved to be a barrier for lifestyle counseling. This caused HCPs to not follow‐up on previously given advice, or to start a treatment even though a patient had failed to improve his lifestyle, “… you compromise your own chances [of a pregnancy]. If you still want to try despite a suboptimal starting situation, well fine, it's not my [IVF] attempt.” (H6). Most HCPs felt reluctant to directly confront their patient about lifestyle, as this could compromise their relationship of trust, “If the patient does not want to talk [about lifestyle], then we don't.” (H2).

##### 3.2.2.2 Lack of uniform care

A common barrier was lack of uniformity on multiple levels of fertility care. Lifestyle policy and counseling differed between fertility centers within the Netherlands and between HCPs within one center. For example, smokers are refused treatment in some Dutch centers. Several HCPs noted that if couples are refused treatment because of their lifestyle, “they would just seek and get treatment elsewhere.” (H6). HCPs also attributed varying levels of importance to different lifestyle factors; recreational drug use and cigarette smoking were considered most important, whereas alcohol consumption received little to no attention. One interviewee mentioned putting minimal effort into lifestyle counseling of patients with a language barrier.

##### 3.2.2.3 Limited and unclear evidence on lifestyle and male fertility

An important barrier for lifestyle counseling was the limited high‐quality clinical evidence on lifestyle and male fertility. The influence of lifestyle on different parameters of male fertility was often unclear to them, which led them to provide less lifestyle counseling to their male patients compared with their female patients. Most HCPs found it challenging to persuade men with a normal semen analysis to make lifestyle improvements, “once the semen analysis is good, lifestyle disappears to the background.” (H3).

##### 3.2.2.4 Interaction between hcp and patient

A patient's emotional, angry, or defensive reaction to lifestyle counseling was a commonly reported barrier. One HCP felt “like I have to walk on eggshells.” (H6), when discussing lifestyle. An additional barrier was that HCPs do not feel trained to motivate their patients. All interviewees noted that men often perceive themselves as less important in the fertility problem. HCPs experienced that men often need to be convinced that they play a significant role and that improving their lifestyle could positively impact their chance to father a (healthy) child.

##### 3.2.2.5 Organization of care

Mainly, HCPs felt that their lifestyle counseling was limited by the organization of care. All interviewees thought that lifestyle counseling would improve if this was also discussed by the general practitioner when consulting a couple before referral. Other barriers were the limited time, “all your time can be consumed by discussing [medical] problems instead of unhealthy habits.” (H2), and limited covering by health insurance, “we used to plan appointments with our nurses for lifestyle support but these expenses cannot be claimed from health insurance.” (H1).

##### 3.2.2.6 Societal factors

The paradigm shift concerning (talking about) lifestyle was a facilitator. When addressing an unhealthy lifestyle, HCPs encountered less resistance compared with a few years ago. Moreover, some viewed that the negative image of smoking in the media had contributed to more patient awareness, “cigarette smoking was once considered a personal choice beyond public comment, but I feel that perception is now changing.” (H3).

### 3.3 Questionnaire

Fifty men fit the eligibility criteria and completed the questionnaire (Table 1).

#### 3.3.1 Rating of Facilitators and Barriers for Lifestyle Change

The facilitators most frequently ranked as most important were: the perception of improved chances of fathering a child (44%) and receiving support from their partner (10%). The most important barriers were stress and a busy life (32%), unhealthy habits tied to social activities (22%), and normal semen quality (18%). The mean ranking scores per facilitator and barrier are listed in Table 2. Distributions per factor can be found in Supporting Information Table S1.

**TABLE 2:** Mean ranking scores^a^ of facilitators and barriers and number of participants that used the factor in their ranking.

Facilitator	Mean rank	Used in ranking *n* (%)	Barrier^b^	Mean rank	Used in ranking *n* (%)
The perception of improved chances of fathering a child	3.32	38 (76)	Stress or a busy life	2.60	30 (60)
The desire to have done everything in my power to fulfill my child wish	1.96	30 (60)	Unhealthy habits are part of social activities	1.70	19 (38)
Improved health of offspring	1.54	23 (46)	Normal semen quality	1.40	17 (34)
That the treatment is a burden for my partner	1.06	20 (40)	Unhealthy lifestyle of peers	0.66	9 (18)
The information provided by my HCP about lifestyle and fertility	0.90	12 (24)	I do not believe that improving my lifestyle will improve my chances to father a child	0.58	9 (18)
The desire to be a good example for future offspring	0.84	14 (28)	Unclear information about lifestyle and fertility	0.42	5 (10)
I am not allowed to start treatment if I do not improve my lifestyle	0.48	8 (16)	Fertility care is focused on my partner	0.30	5 (10)
Impaired semen quality	0.40	8 (16)	My peers do not know about our fertility treatment	0.28	5 (10)
Support from peers	0.26	6 (12)	A coercive HCP	0.20	3 (6)
Benefits for general health	0.26	4 (8)	No personal interaction with my HCP	0.18	3 (6)
The start of fertility treatment	0.16	5 (10)	A long and disappointing fertility journey	0.16	4 (8)
The personal interaction with my HCP	0.14	3 (6)	Fear of the reaction of peers	0.16	2 (4)
			Insufficient information about lifestyle and fertility	0.14	2 (4)
			No support from partner	0	0 (0)

^a^ The highest possible ranking score is 5 (indicating high importance) and the lowest possible ranking score is 0 (indicating low importance).

^b^ Eleven patients (22%) indicated that they experienced no barriers in improving their lifestyle.

#### 3.3.2 Patient‐Centeredness and Intention to Change

Table 3 shows the frequencies per response option of the modified PCQ‐I questionnaire and the intention to change scale. The results from the modified PCQ‐I showed that the majority of patients received sufficient and clear information on lifestyle, which was often tailored to their personal situation. A small proportion experienced no room for their questions and opinions. Almost three‐quarters of the patients was likely or very likely to improve their lifestyle in the coming 6 months.

**TABLE 3:** Questionnaire outcomes per item of modified PCQ‐I and intention to change.

Modified PCQ‐I questionnaire				
	Scale, *n* (%)^a^			
Item on questionnaire	No	Yes, but not enough	Yes, sufficient	Yes, extensive
Did you receive general information about lifestyle and male fertility?	2 (4)	2 (4)	30 (60)	16 (32)
Did you receive personal information tailored to your situation?	13 (26)	4 (8)	22 (44)	7 (14)
Did your HCP ask about your lifestyle during follow‐up appointments?	12 (24)	3 (6)	7 (14)	3 (6)

	No	A little	Mostly	Totally
Was the information you received clear?	2 (4)	1 (2)	8 (16)	37 (74)
Was there room to ask questions to your HCP about your lifestyle?	3 (6)	5 (10)	6 (12)	31 (62)
Was your HCP open to your opinion and ideas about your lifestyle?	2 (4)	5 (10)	6 (12)	12 (24)

	No, but I would have wanted this	No, and I did not want this	Yes
Did your HCP refer you to another HCP to support you in changing your lifestyle?	3 (6)	35 (70)	8 (16)
Did your HCP or other fertility HCPs actively support you in changing your lifestyle?	4 (8)	38 (76)	3 (6)

Intention to change, *n* (%) (I will improve my lifestyle in the coming 6 months)				
Very unlikely	Unlikely	Neutral	Likely	Very likely
0 (0)	3 (6)	10 (20)	17 (34)	20 (40)

^a^ Some values in the modified PCQ‐I questionnaire are missing, as participants answered “I do not know” or “not applicable.” This means that not all numbers add up to 50, and that not all percentages add up to 100.

We found that there was no correlation between duration of infertility or male age and intention to change (*r* = 0.24, 95% confidence interval −0.04 to 0.48, *p* = 0.10; *r* = 0.08, 95% confidence interval −0.20 to 0.35, *p* = 0.56, respectively). There were no significant differences in intention to change between different fertility diagnoses (*p* = 0.96), BMI ≥ 25 or < 25 (*p* = 0.26), alcohol consumption (*p* = 0.08), smoking status (*p* = 0.08), or recreational drug use (*p* = 0.85).

## 4 Discussion

In this mixed‐methods study, we found that the most important facilitators for lifestyle change in men seeking fertility care are the wish to improve their chances to father a child and their partner's support. The most important barriers are stress, a busy life, an unhealthy lifestyle being part of social activities, and normal semen quality.

Fertility HCPs’ lifestyle counseling was influenced by facilitators such as professional responsibility and the changing societal lifestyle paradigm, and by barriers such as limited high‐quality evidence about male lifestyle and fertility, time limitations, and lack of uniform care.

Up to now, studies on lifestyle changes in fertility patients have mainly focused on women. In a recent review, only 11 out of 27 papers included men, often in small numbers [34]. Moreover, these studies were mostly quantitative or addressing the effect of an intervention [35, 36, 37, 38, 39, 40].

Comparing our study to others shows that men and women share similar perspectives on the factors influencing lifestyle change. Both consider the support of their partner, peers, and HCP as an important facilitator to change their lifestyle [34, 35, 40].

The findings of this study are in line with previous research on factors involved in lifestyle counseling by fertility HCPs. Professional responsibility was previously described as an enabler for lifestyle counseling by Boedt et al. [41]. However, the barrier of the patient's own responsibility is new. Additionally, we are the first to find that limited evidence on male lifestyle and fertility hinders HCPs’ lifestyle counseling. This weakens information given to patients, which was also acknowledged by patients in our study.

One strength of this study is that it is the first to explore which factors enable and hinder men seeking fertility care to change their lifestyle. Our mixed‐methods approach made it possible to obtain rich and authentic perspectives and to subsequently show which factors to prioritize in medical practice. Adding the experience of fertility HCPs enhances our understanding of challenges in lifestyle counseling of these patients.

A limitation of this study is the small number of interviewed HCPs, although data saturation was reached. This study was conducted in the Netherlands, where public insurance covers fertility care and some lifestyle interventions. Furthermore, the sale of soft drugs such as cannabis is tolerated in the Netherlands. Around 20% of Dutch adults had smoked cigarettes and 77% had consumed alcohol in 2023 [42, 43]. This limits the study's generalizability to countries with different (insurance) policies and social norms and practices related to lifestyle.

This study may help patients in multiple ways. HCPs should emphasize the role of the partner by discussing how the couple can achieve a durable lifestyle change together. To patients with a normal semen quality, HCPs should stress the effect of lifestyle on pregnancy loss and the offspring. A lifestyle coach or dedicated nurse could help identify a patient's facilitators and barriers to ensure a more comprehensive and tailored approach. Limited evidence in couples and women shows that an online lifestyle program reduces unhealthy behavior [44, 45]. Furthermore, reviews on smoking cessation show that a nursing intervention and individual behavioral counseling increase tobacco abstinence [46, 47].

To support fertility HCPs, more evidence is needed on male lifestyle and its influence on fertility, pregnancy loss, and offspring. Robust studies on the influence of drug and alcohol consumption would add to the current body of evidence. Information and protocols on lifestyle and fertility care should be uniform, within one center at least, but preferably nationally.

## 5 Conclusion

Important facilitators for lifestyle improvement by men seeking fertility care are the wish to improve their chances to father a child and their partner's support. The most important barriers are stress, a busy life, an unhealthy lifestyle being part of social activities, and normal semen quality. Professional responsibility and changing societal lifestyle paradigm enable HCPs to discuss lifestyle with male patients. Barriers reported by HCPs are the lack of evidence on this topic, time constraints, and lack of uniform care. The results from this study should be used to inspire fertility departments to (re)design lifestyle interventions and policies for male patients.

## Author Contributions

Each author has contributed substantially to this article. Wiep R. de Ligny has contributed to the conception and design of the work, as well as the collection, analysis, and interpretation of the data, and drafting the final article. K. Fleischer, D. D. M. Braat, J. P. de Bruin, and I. L. Abma were involved in the study design, the interpretation of the data, and revision and final approval of the article. H. Grens has contributed to the analysis and interpretation of the data.

## Conflicts of Interest

The authors declare no conflicts of interest.

## Supporting information




**Table S1**: Marginal distributions of patient‐reported facilitators and barriers.

## Data Availability

Data from this research are stored under closed conditions in a Data Acquisition Collection (DAC: https://doi.org/10.34973/ve42‐ew24). Data are stored under closed conditions as the content of the interviews could be traceable to a specific person. Sharing of the data can be requested through the corresponding author.
