# Supplementary material for: What Moves Men to Change? A Mixed‐Methods Study on Facilitators and Barriers of Lifestyle Changes in Men Seeking Fertility Care
Source: Andrology. 2025 Oct 26;14(5):1220–30. doi: 10.1111/andr.70133 (PMC13266432; doi:10.1111/andr.70133)
Supplement: Supplementary file 1 — Table S1: Marginal distributions of patient‐reported facilitators and barriers. [file ANDR-14-1220-s001.docx]

Supplementary Table 1. Marginal distributions of patient-reported facilitators and barriers.

| **Facilitator** | **1^st^** | **2^nd^** | **3^rd^** | **4^th^** | **5^th^** | **Barrier** | **1^st^** | **2^nd^** | **3^rd^** | **4^th^** | **5^th^** |
| --- | --- | --- | --- | --- | --- | --- | --- | --- | --- | --- | --- |
| The perception of improved chances of fathering a child | 22 | 11 | 3 | 1 | 1 | Stress or a busy life | 17 | 10 | 1 | 0 | 2 |
| The desire to have done everything in my power to fulfill my child wish | 4 | 11 | 7 | 5 | 3 | Unhealthy habits are part of social activities | 11 | 6 | 2 | 0 | 0 |
| Improved health of offspring | 3 | 10 | 2 | 8 | 0 | Normal semen quality | 9 | 5 | 0 | 2 | 1 |
| Support from partner | 5 | 4 | 4 | 2 | 1 | No barriers | 4 | 0 | 0 | 0 | 0 |
| That the treatment is a burden for my partner | 3 | 2 | 5 | 5 | 5 | Unhealthy lifestyle of peers | 3 | 2 | 3 | 0 | 1 |
| The information provided by my HCP about lifestyle and fertility | 4 | 3 | 3 | 2 | 0 | I do not believe that improving my lifestyle will improve my chances to father a child | 1 | 2 | 4 | 2 | 0 |
| The desire to be a good example for future offspring | 2 | 3 | 3 | 5 | 1 | Unclear information about lifestyle and fertility | 3 | 1 | 0 | 1 | 0 |
| I am not allowed to start treatment if I do not improve my lifestyle | 2 | 0 | 4 | 0 | 2 | Fertility care is focused on my partner | 0 | 2 | 2 | 0 | 1 |
| Impaired semen quality | 1 | 0 | 4 | 0 | 3 | My peers do not know about our fertility treatment | 1 | 0 | 2 | 1 | 1 |
| Support from peers | 0 | 0 | 3 | 1 | 2 | My HCP is too coercive | 0 | 0 | 2 | 1 | 0 |
| Benefits for general health | 2 | 0 | 0 | 1 | 1 | No personal interaction with my HCP | 1 | 0 | 0 | 2 | 0 |
| The start of fertility treatment | 0 | 0 | 1 | 1 | 3 | A long and disappointing fertility journey | 0 | 0 | 1 | 2 | 1 |
| The personal interaction with my HCP | 1 | 0 | 2 | 0 | 0 | Fear of the reaction of peers | 0 | 2 | 0 | 0 | 0 |
|  |  |  |  |  |  | Insufficient information about lifestyle and fertility | 0 | 1 | 1 | 0 | 0 |
|  |  |  |  |  |  | No support from partner | 0 | 0 | 0 | 0 | 0 |
| Total | 50 | 44 | 41 | 31 | 22 | Total | 50 | 31 | 18 | 11 | 7 |
